# Takotsubo cardiomyopathy concurrent with hyperthyroidism following COVID-19—a case report and literature review

**DOI:** 10.3389/fcvm.2026.1862540

**Published:** 2026-07-08

**Authors:** Liya Lin, Shanshan Liu, Yi Fang, Qijing Zhou, Minyue Jia, Hanxiao Yu, Kai Cheok, Xuehua Zhang, Lingxia Ye, Xiaoxiao Song

**Affiliations:** 1 Center for Basic Research, Second Affiliated Hospital, School of Medicine, Zhejiang University, Hangzhou, Zhejiang, China; 2 Department of Cardiology, The Second Affiliated Hospital, Zhejiang University School of Medicine, Hangzhou, Zhejiang, China; 3 Department of Psychiatry and Behavioral Sciences, University of California, San Francisco, CA, United States; 4 Department of Radiology, Second Affiliated Hospital, Zhejiang University School of Medicine, Hangzhou, Zhejiang, China; 5 Department of Ultrasound, The Second Affiliated Hospital School of Medicine, Zhejiang University School of Medicine, Hangzhou, Zhejiang, China; 6 Department of Medicine, Zhejiang University, Hangzhou, Zhejiang, China; 7 Department of Endocrine and Metabolic Diseases, The Second Affiliated Hospital, Zhejiang University School of Medicine, Hangzhou, Zhejiang, China

**Keywords:** COVID-19, ECG, hyperthyroidism, SARS-CoV-2, takotsubo cardiomyopathy

## Abstract

**Background:**

The convergence of viral infection, autoimmune thyroid disease, and cardiovascular vulnerability may create a “triple systemic stress” predisposing to Takotsubo cardiomyopathy (TTS). We describe a case illustrating this potential pathophysiological triad.

**Case presentation:**

A previously healthy 21-year-old male presented with sinus tachycardia, anterior ST-segment elevation on preoperative Electrocardiogram (ECG), and regional wall motion abnormalities on echocardiography two weeks after SARS-CoV-2 infection. Emergency coronary angiography ruled out myocardial infarction and coronary stenosis. Clinical features suggested thyrotoxicosis, confirmed by biochemical and imaging studies. Cardiac magnetic resonance demonstrates subendocardial hypoperfusion in the interventricular septum accompanied by abnormal myocardial motion. Treatment with methimazole and beta-blockers led to resolution of cardiac dysfunction and gradual normalization of thyroid function.

**Conclusion:**

This case report describes a temporal association among SARS-CoV-2 infection, exacerbation of autoimmune thyroid dysfunction, and Takotsubo syndrome. The virus may contribute to Graves' disease exacerbation through immune-inflammatory mechanisms, while potentially inducing myocardial stress via cytokine-mediated pathways. These converging factors—combined with thyrotoxicosis-related catecholamine sensitivity—may act synergistically to increase TTS susceptibility. Clinically, thyroid evaluation may be considered in COVID-19 patients presenting with cardiac symptoms, and an integrated management approach addressing viral, endocrine, and cardiovascular aspects could be beneficial. The precise interconnections among these factors remain speculative and warrant further investigation in larger cohorts.

## 1 Introduction

Takotsubo cardiomyopathy (TTS), also known as stress-induced cardiomyopathy, apical ballooning syndrome, or broken heart syndrome, is characterized by transient wall-motion abnormalities of the left or both ventricles. First described in Japanese patients in 1983 by Dote et al., the condition presents with acute myocardial infarction-like symptoms but exhibits a distinctive left ventricular contraction pattern resembling a takotsubo—a traditional Japanese octopus trap with a round bottom and narrow neck. Recognition of TTS has grown substantially since its initial report. It was formally acknowledged by the American Heart Association (AHA) in 2006 and later classified as a distinct primary cardiomyopathy in 2008; it is now included in major biomedical databases such as the National Center for Biotechnology Information (NCBI) (1).

Acute TTS often mimic acute coronary syndrome (ACS), manifesting with electrocardiographic abnormalities (ST-segment elevation or depression, left bundle branch block, T-wave inversion, prolonged corrected QT interval), elevated B-type natriuretic peptide, and troponin elevation. Diagnosis remains one of exclusions, relying principally on imaging modalities including echocardiography, cardiac magnetic resonance (CMR), and coronary angiography. During the acute phase, echocardiography typically demonstrates segmental left ventricular dysfunction; CMR may reveal focal perfusion deficits and late gadolinium enhancement, whereas coronary angiography shows no obstructive coronary artery disease. Characteristically, the ventricular dysfunction is reversible and frequently resolves within weeks to months (2, 3). Formal diagnostic criteria, such as the Revised Mayo Clinic Criteria, emphasize: 1) transient regional wall-motion abnormalities of the left ventricle (rarely the right ventricle); 2) absence of obstructive coronary disease or acute plaque rupture on angiography; 3) new electrocardiographic abnormalities (ST-segment elevation/depression, T-wave inversion) or modest elevation in cardiac troponin; and 4) exclusion of other causes such as pheochromocytoma or myocarditis (4).

This case report describes a patient who developed concurrent hyperthyroidism and TTS following SARS-CoV-2 infection. Although the patient had a family history of hyperthyroidism, there was no personal history or pre-infection symptomatology. The temporal association raises the possibility that hyperthyroidism was precipitated or exacerbated by the viral infection. Emerging evidence suggests that SARS-CoV-2 can directly infect thyroid follicular cells through ACE2 receptors and indirectly disrupt thyroid function via systemic inflammatory responses or cytokine storms. Although catecholamine levels in this patient were within the normal range, the pathogenesis of TTS in this setting likely involves a multifactorial interplay: direct myocardial injury from SARS-CoV-2-related inflammation, hemodynamic and inotropic/chronotropic effects of hyperthyroidism, and increased myocardial sensitivity to catecholamines induced by thyrotoxicosis. The present case aims to explore the potential interrelationship among COVID-19, hyperthyroidism, and TTS.

## 2 Case presentation

A 21-year-old man was admitted to the hospital after an abnormal electrocardiogram (ECG) was noted during a preoperative evaluation for the excision of a cutaneous pigmented nevus. He reported experiencing palpitations for several weeks, for which he had not previously sought medical attention. The initial ECG demonstrated sinus tachycardia and ST-segment elevation in multiple leads (Figure 1A). The patient denied chest pain or dyspnea. Approximately two weeks prior to admission, he had developed fever and diarrhea, tested positive for SARS-CoV-2, and noted persistent palpitations. He also reported an intermittent dry cough, excessive sweating, and an approximate 7.5 kg weight loss over the preceding three months. On admission, vital signs were as follows: temperature 37.2 °C, heart rate 144 bpm, respiratory rate 19 breaths/min, blood pressure 150/67 mmHg, and SpO₂ 99%. Physical examination revealed a grade III goiter, an accentuated S2 heart sound, and coarse breath sounds bilaterally. No other significant abnormalities were detected.

**Figure 1:**
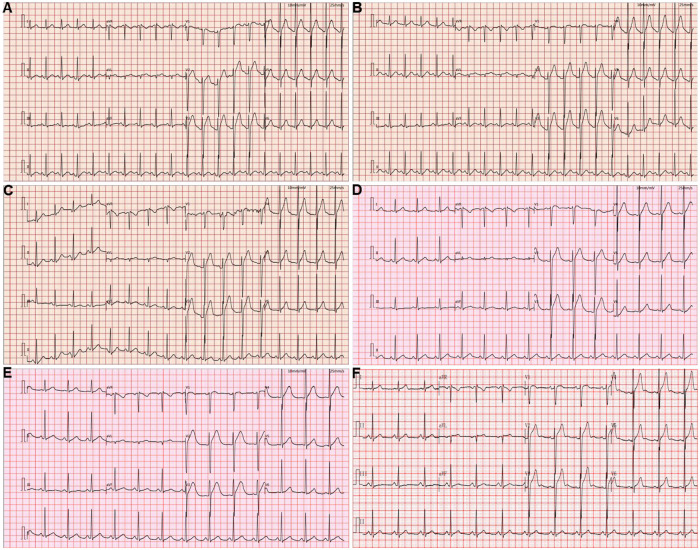
Electrocardiographic evolution. **(A)** Day 1 (admission, 14:42): Sinus tachycardia (122 bpm) with diffuse anterior ST-segment elevation in leads V1-V6 (red arrows, maximal in V2-V4), mimicking acute anterior myocardial infarction; note the absence of reciprocal ST depression. **(B)** Day 1 (2 h later, 16:28): Persistent ST elevation with QT interval prolongation (green bracket) and early T-wave inversion in V5-V6 (yellow arrow). **(C)** Day 2: ST elevation territory shrinks to anterolateral leads (orange arrows), with heart rate reduction to 102 bpm. **(D)** Day 6: Marked ST resolution with only mild residual elevation in V1-V2 (green arrows) and appearance of left ventricular voltage criteria (blue arrows, RV5 3.595 mV). **(E)** Day 9: Complete ST normalization (green checkmarks). **(F)** Day 20: Fully normalized electrocardiogram without Q waves or persistent repolarization abnormalities, with isolated LV voltage criteria.

### 2.1 Diagnostic assessment

Day 1:

- The ECG of admission at 14:42 showed Sinus tachycardia (122 bpm) with diffuse anterior ST-segment elevation in leads V1-V6 (red arrows, maximal in V2-V4), mimicking acute anterior myocardial infarction; note the absence of reciprocal ST depression (Figure 1A). 2 h later at 16:28, a repeated ECG showed persistent ST elevation with QT interval prolongation (green bracket) and early T-wave inversion in V5-V6 (yellow arrow) (Figure 1B).
- Initial echocardiography showed tachycardia (HR 128 bpm) and a left ventricular ejection fraction (LVEF) of 55.7% (biplane Simpson's). Although myocardial thickness was normal, regional hypokinesis was noted in the basal-to-mid inferoseptum and the basal inferior wall (ASE 17-segment model; Supplementary Video S1), suggesting mildly impaired regional contractility.
- Laboratory Findings：N-terminal pro-brain natriuretic peptide (NT-proBNP) was 38 pg/mL, troponin was 0.009 ng/mL (within normal range), and creatine kinase-MB (CK-MB) was 14 U/L.

Day 2:

- The ECG on day 2 showed ST elevation territory shrinks to anterolateral leads (orange arrows), with heart rate reduction to 102 bpm (Figure 1C).
- Coronary angiography revealed approximately 20% stenosis in the mid segment of the left anterior descending artery with associated myocardial bridging; no obstructive coronary artery disease was identified (Supplementary Figure S1).

Day 3:

Laboratory Findings identified significant thyroid dysfunction: elevated total triiodothyronine (T3 > 9.22 nmol/L, reference range: 0.98 nmol/L-2.33 nmol/L), free triiodothyronine (FT3 > 30.72 pmol/L, reference range: 2.43 pmol/L-6.01 pmol/L), total total thyroxine (T4 = 252.2 nmol/L，reference range: 62.7 nmol/L-150.8 nmol/L) and free thyroxine (FT4 = 51.76 pmol/L，reference range: 9.01pmol/L-19.05pmol/L), accompanied by suppressed thyroid-stimulating hormone (TSH <0.01 mIU/L, referencerange: 0.35mIU/L-4.94mIU/L), and elevated thyrotropin receptor antibody (TRAb = 10.98 IU/L, reference range: <1.75 IU/L). Catecholamine and metabolite levels were within normal limits. Renin levels were elevated, and serum potassium was 3.43 mmol/L.

Day 5:

- Cardiac Magnetic Resonance demonstrates subendocardial hypoperfusion in the interventricular septum accompanied by abnormal myocardial motion, but no significant abnormal signals were observed on either dark-blood sequences or late gadolinium enhancement imaging (Figure 2). These findings confirmed involvement of the interventricular septum, suggestive of an acute myocardial injury pattern.
- Thyroid Imaging: ultrasound suggested diffuse thyroid disease (Figure 3A); scintigraphy showed enlarged thyroid with increased technetium-99 m pertechnetate uptake (14.7%), supporting the diagnosis of hyperthyroidism (Figure 3B).

**Figure 2:**
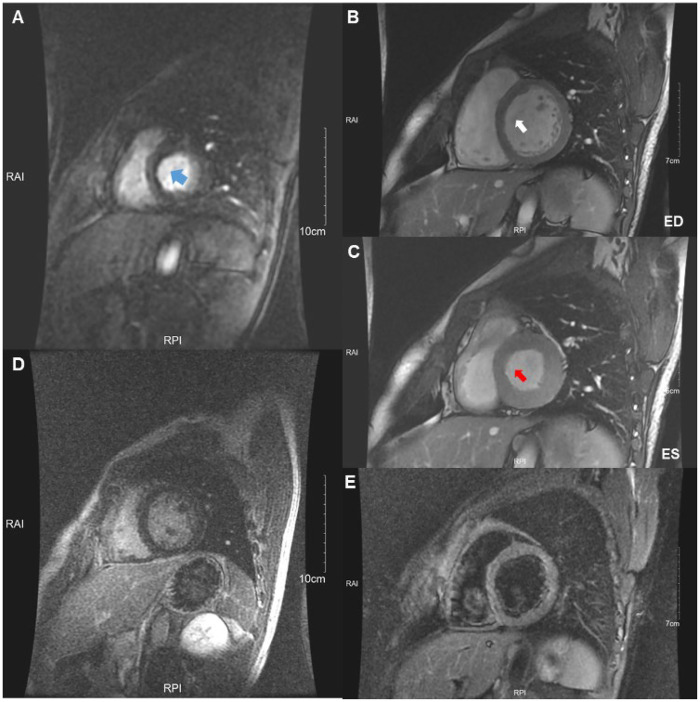
(1) On first-pass perfusion image **(A)** (blue arrow), hypoperfusion is noted in the mid-anterior interventricular septum. (2) On cine sequences [Images **(B)** and **(C)**], abnormal myocardial motion of the interventricular septum is demonstrated (white arrow: relatively thickened myocardium in diastole; red arrow: relatively thinned myocardium in systole). (3) No obvious hyperintensity is seen on the black-blood sequence [Image **(D)**], and no late gadolinium enhancement (LGE) is present [Image **(E)**], excluding myocardial infarction and myocarditis.

**Figure 3:**
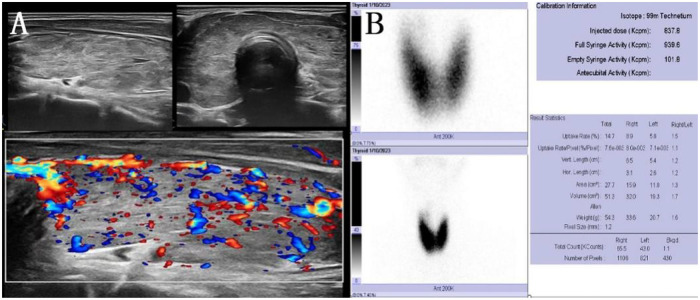
**(A)** Thyroid color Doppler ultrasound showing diffuse thyroid lesions with an “inferno sign”. **(B)** Thyroid scintigraphy reveals bilateral thyroid enlargement with diffusely increased technetium-99 m pertechnetate uptake, consistent with hyperthyroidism.

Day 6:

The ECG on day 6 showed marked ST resolution with only mild residual elevation in V1-V2 (green arrows) and appearance of left ventricular voltage criteria (blue arrows, RV5 3.595 mV) (Figure 1D).

Day 7:

Myocardial Perfusion/Metabolism Imaging (Dual-Isotope Simultaneous Acquisition, DISA) showed no significant abnormalities, effectively ruling out transmural infarction (Supplementary Figure S2).

Day 8:

Repeat echocardiography revealed normal myocardial thickness and the disappearance of wall motion abnormalities (LVEF 63.5%)

Day 9:

The ECG on day 9 showed complete ST normalization (green checkmarks) (Figure 1E).

Day 20:

The ECG on day 20 showed Fully normalized electrocardiogram without Q waves or persistent repolarization abnormalities, with isolated LV voltage criteria (Figure 1F).

### 2.2 Treatment

Initially, the patient was managed for suspected acute myocardial infarction and received aspirin for antiplatelet therapy, atorvastatin for lipid-lowering and plaque stabilization, coenzyme Q10 for myocardial protection, and combined ivabradine and propranolol for heart rate control. Following coronary angiography that excluded obstructive coronary artery disease, coupled with confirmed Graves' disease-induced hyperthyroidism, the diagnosis was revised to stress cardiomyopathy complicated by hyperthyroidism, methimazole 15 mg daily was initiated for hyperthyroidism management.

### 2.3 Outcome and follow-up

Post-treatment, he heart rate normalized to 80–85 bpm. Although serial ECGs ST resolution with only mild residual elevation in V1-V2 on day 6 (Figure 1C), sinus rhythm was stable by day 9 (Figure 1D). Repeat echocardiography (day 8) revealed normal myocardial thickness and the disappearance of wall motion abnormalities (LVEF 63.5%). Repeated ECG on day 20 showed fully normalized electrocardiogram without Q waves or persistent repolarization abnormalities, with isolated LV voltage criteria (Figure 1F). All cardiac structures and flow parameters returned to within normal limits. At the seven-month follow-up, the patient remained asymptomatic with a preserved LVEF of 65.1%, indicating a full recovery of myocardial function.

At the one-month follow-up, thyroid function showed improvement, although TSH remained suppressed. By two months, ECG and echocardiographic findings had normalized, and thyroid function had further improved, with FT3 at 9.01 pmol/L, FT4 at 23.06 pmol/L, and TSH < 0.01 mIU/L. Methimazole was continued with gradual dose reduction. By the sixth month, T3, T4, and TSH had all returned to normal levels, though TRAb remained mildly elevated at 5.14 IU/L. During the two-and-a-half-year follow-up, thyroid function had gradually normalized and TRAb had turned negative, leading to the discontinuation of methimazole. However, six months after withdrawal, T3 and T4 were again moderately elevated, accompanied by an increase in TRAb to 9.19 IU/L and a recurrent decline in TSH, prompting reinstitution of methimazole at a maintenance dose of 10 mg/day. Notably, despite the recurrence of hyperthyroidism, echocardiographic findings remained normal (Supplementary Figure S4), providing further support for the absence of structural myocardial involvement.

This case highlights the complex interplay between thyroid dysfunction and stress-induced cardiac injury. The patient's hyperthyroidism likely contributed to tachycardia and increased myocardial oxygen demand, precipitating stress cardiomyopathy in the context of recent SARS-CoV-2 infection. Long-term management required careful balance of cardiac support and antithyroid therapy, underscoring the importance of interdisciplinary follow-up in such cases.

## 3 Discussion

We report a unique case of a young male patient who subsequently developed hyperthyroidism and Takotsubo cardiomyopathy (TTS) following recent SARS-CoV-2 infection. The patient's clinical course presented a diagnostic challenge: incidental identification of sinus tachycardia with extensive anterior ST-segment elevation on a preoperative electrocardiogram (ECG) prompted a comprehensive workup, which confirmed transient regional wall-motion abnormalities and myocardial injury. Conversely, coronary angiography definitively ruled out ischemic etiologies, including acute coronary syndrome. Notably, despite the absence of significant elevations in cardiac troponin or myocardial enzyme levels, both ECG abnormalities and echocardiographic findings resolved completely within 8 days of admission. This recovery preceded effective control of the hyperthyroid state, which was characterized by markedly elevated total and free thyroid hormones (TT3, TT4, FT3, FT4), suppressed thyroid-stimulating hormone (TSH), positive thyroid autoantibodies, and increased diffuse uptake on technetium-99 m pertechnetate thyroid scintigraphy. More compellingly, during a two-and-a-half-year follow-up, no recurrence of echocardiographic or ECG abnormalities was observed, even during a subsequent episode of hyperthyroidism that occurred after discontinuation of antithyroid medication.

Given the temporal relationship with SARS-CoV-2 infection, COVID-19-associated myocarditis represented a critical alternative diagnosis. Myocarditis was rigorously excluded based on the updated Lake Louise Criteria (Ferreira et al., JACC Cardiovasc Imaging 2018). cardiac magnetic resonance provided more precise anatomical localization, demonstrating focal subendocardial hypoperfusion and severe hypokinesis (systolic thickening less than 30%) confined to the anteroseptal mid-ventricular segment (segment 7), with normal contractility (thickening greater than 50%) in adjacent segments. Notably, T2 dark-blood imaging showed no signal elevation and late gadolinium enhancement was completely absent, excluding myocardial infarction and myocarditis. The CMR findings did not meet the updated Lake Louise criteria for acute myocarditis and were suggestive of no active myocarditis. The rapid resolution of ventricular dysfunction (8 days) and characteristic perfusion-contraction mismatch further distinguished this case from myocarditis, which typically demonstrates slower recovery and persistent LGE (5). Although several anatomical TTS variants have been described four major types can be differentiated based on the distribution of regional wall motion abnormalities. The most common TTS type and widely recognized form is the (i) apical ballooning type also known as the typical TTS form, which occurs in the majority of cases. Over the past years, atypical TTS types have been increasingly recognized. These include the (ii) midventricular, (iii) basal, and (iv) focal wall motion patterns. Recently, it has been demonstrated that patients suffering from atypical TTS have a different clinical phenotype (3).

The normal cardiac troponin in this case, despite extensive wall-motion abnormalities, reflects the characteristic “disconnect” of TTS. Troponin elevation occurs in most TTS cases but may be normal in 10%–20%, particularly with early presentation (<6 h), reverse variant (smaller myocardial stunning area), or rapid resolution phase. The modest biomarker elevation relative to wall-motion extent distinguishes TTS from myocardial infarction or myocarditis, where troponin correlates with necrotic extent. CMR confirmation of absent LGE supported viable myocardium with transient stunning rather than necrosis (6, 7). Although cardiac troponin is typically elevated in TTS, our patient presented with normal levels. This may be due to early presentation (<6 h), the reverse/basal variant, or the characteristic biomarker-wall motion mismatch in TTS. Unlike acute coronary syndrome, the mild troponin elevation in TTS reflects catecholamine-mediated myocardial stunning rather than extensive necrosis. The absence of clinically significant myocardial necrosis is further confirmed by CMR showing absence of gadolinium hyperenhancement (LGE-negative), consistent with viable myocardium with transient contractile dysfunction (8). The patient's Burch-Wartofsky score of 40 points indicates impending thyroid storm rather than true crisis, suggesting that the severe thyrotoxicosis may have contributed to TTS through a different pathophysiological mechanism.

Takotsubo syndrome, first described in 1990, is classically characterized by transient apical ballooning with female predominance (9). Multiple morphological variants are recognized, including mid-ventricular, basal, and focal subtypes (10). The classic apical variant accounts for approximately 80%–85% of all TTS cases (11). The present case represents the rare reverse (basal) variant, defined by predominant wall-motion abnormalities involving the basal to mid-ventricular segments with preserved apical contractility, reported to constitute only 2%–2.2% of all TTS patients (10–12). This atypical presentation poses a significant risk of misdiagnosis, as its ECG and biomarker profiles may closely mimic acute coronary syndrome, while its regional wall-motion pattern deviates from the classic apical phenotype. The pathophysiological mechanisms underlying basal-type TTS remain incompletely understood but are thought to be associated with regional heterogeneity in catecholamine sensitivity and *β*-adrenergic receptor distribution across the left ventricular myocardium. Consistently, elevated catecholamine concentrations during the acute phase of TTS are recognized as a direct driver of myocardial injury and coronary vasoconstriction (13).

Although the pathogenesis of TTS is not fully elucidated, its hallmark is transient left ventricular dysfunction triggered by emotional or physical stressors. Neuroendocrine dysregulation, particularly a surge in catecholamines, is considered central to this process. Excessive catecholamines may induce myocardial injury through multiple potential mechanisms, including direct cardiotoxicity, adrenoceptor overstimulation, coronary microvascular spasm, and increased cardiac workload. Additionally, endothelial dysfunction associated with changes in sex hormone levels has been proposed as a contributing mechanism, which may explain the higher susceptibility in postmenopausal women (14, 15). Within this framework, the COVID-19 pandemic has provided a unique context for understanding TTS triggers. Studies have shown a significant increase in TTS incidence during the pandemic. A large cohort study analyzed 1,914 patients presenting with acute coronary syndrome-like symptoms found that the TTS diagnosis rate increased from 1.5%–1.8% pre-pandemic to 7.75% during the pandemic (16), and recent reports have confirmed TTS as a complication of COVID-19 (17–20). This proportion was even higher among COVID-19-positive patients, with studies reporting concurrent TTS in 2% to 4.2% of such cases. This data strongly suggests a potential association between COVID-19 and TTS. The increased incidence can be attributed to multiple mechanisms (21). First, viral infection can trigger an excessive immune response (“cytokine storm”), directly causing myocardial injury. Second, infection-related physiological stress and inflammation can activate the sympathetic nervous system, leading to a catecholamine surge. Third, the COVID-19-associated hypercoagulable state can promote microthrombosis and microvascular dysfunction. Beyond biological mechanisms, the psychosocial stressors prevalent during the pandemic—widespread fear, anxiety, grief, and economic distress—collectively elevated public levels of psychological stress, which may further increase TTS risk by activating the sympathetic-adrenal axis.

Excessive catecholamines and sympathetic overactivity are central to TTS pathogenesis, and thyrotoxicosis is a recognized predisposing factor. According to a 2022 review, at least 25 cases of stress-induced cardiomyopathy associated with thyrotoxicosis have been reported, approximately half related to Graves' disease and half to exogenous causes (22). The association is rooted in the profound interdependence between thyroid hormones and the adrenergic axis. Thyroid hormones may potentiate catecholamine-mediated myocardial injury through synergistic effects: in hyperthyroidism, excessive hormones upregulate *β*-adrenergic receptor density, promote G protein overexpression, and modulate downstream signaling, thereby increasing myocardial sensitivity to catecholamines and lowering the threshold for catecholamine-induced injury—key elements in TTS pathogenesis (23, 24). However, the myocardial effects induced by thyroid hormones are not solely dependent on *β*-adrenergic receptor activation. Experimental data indicate that even gene-knockout mice lacking all three *β*-adrenergic receptor subtypes still exhibit cardiovascular responses to hyperthyroidism (25), suggesting that thyroid hormones may also act directly on the cardiovascular system through non-adrenergic pathways. Multiple studies have confirmed that triiodothyronine (T3) can directly enter cardiomyocyte nuclei, bind to thyroid hormone receptors, and regulate the expression of a series of key genes: upregulating sarcoplasmic/endoplasmic reticulum calcium ATPase (SERCA2) and *α*-myosin heavy chain (*α*-MHC), while downregulating genes such as *β*-myosin heavy chain (*β*-MHC), thereby directly altering myocardial contractility, calcium cycling, and diastolic function (26, 27). Furthermore, T3 directly influences cardiac electrophysiology and reduces systemic vascular resistance. In summary, thyroid hormones create a susceptible cardiac milieu for TTS through dual mechanisms: sensitizing the heart to catecholamines via enhanced *β*-adrenergic signaling and altering the basal cardiac state through direct genomic effects on contractility, electrophysiology, and hemodynamics. This dual action constitutes a “susceptible matrix,” making the heart more likely to exceed the TTS threshold when encountering a strong exogenous stressor like COVID-19, explaining this patient's unique clinical course.

As discussed, hyperthyroidism creates a cardiac milieu susceptible to TTS via catecholamine sensitization and direct myocardial effects. We hypothesize that COVID-19 infection may act as a “second hit” on this susceptible substrate, potentially exacerbating the state through effects on thyroid function. The association between COVID-19 and thyroid dysfunction is supported by several mechanisms. First, SARS-CoV-2 utilizes ACE2 and TMPRSS2 as entry points, both highly expressed in thyroid tissue. Viral RNA has been detected in thyroid tissue, and direct pituitary damage may disrupt TSH-secreting cells and pituitary-thyroid axis feedback (28–30). Second, the excessive immune response induced by COVID-19 can indirectly damage the thyroid. Damage-associated molecular patterns (DAMPs) activate innate immune signals, triggering a pro-inflammatory cascade involving IL-6, IFN-*γ*, and T lymphocyte subsets (30, 31).

Emerging evidence suggests that SARS-CoV-2 may contribute to thyroid dysfunction through potential pathways: ACE2-mediated direct infection, cytokine-mediated inflammation, and pituitary-thyroid axis disruption. A meta-analysis of 30 studies (9,707 patients) reported 15% prevalence of thyroid dysfunction in COVID-19, correlating with disease severity (6.2% mild-to-moderate vs. 20.8% severe-to-critical) (19, 32). Abnormal thyroid function was associated with a 3.77-fold increased risk of severe COVID-19, and abnormal TSH levels were linked to potentially higher mortality (33). Case reports provide additional evidence: a pregnant patient with hydatidiform mole presented concurrently with thyroid storm and SARS-CoV-2 infection, ultimately diagnosed with TTS. Researchers proposed that hCG-TSH receptor cross-reactivity induced thyroid storm, with superimposed COVID-19 potentially contributing to TTS precipitation (34). However, the patient's family history of hyperthyroidism suggests underlying autoimmune predisposition independent of COVID-19. We hypothesize that COVID-19 may have acted as a potential trigger on this susceptible substrate but acknowledge that: (1) temporal sequence does not establish causality; (2) absence of elevated catecholamines challenges the classic catecholamine-surge hypothesis; (3) single-case observations cannot be generalized (Figure 4).

**Figure 4:**
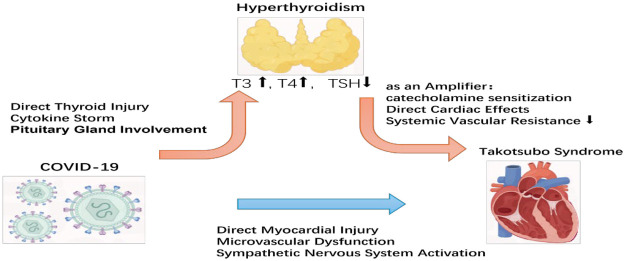
Hypothetical model of potential interactions Among SARS-CoV-2 infection, hyperthyroidism, and takotsubo syndrome. This schematic illustrates proposed pathogenic pathways that may connect COVID-19 to the development of stress-induced cardiomyopathy, with hyperthyroidism potentially acting as a contributing factor. This model is based on a single-case observation and is intended to be hypothesis-generating rather than definitive.

In summary, this case report describes a temporal association among COVID-19, hyperthyroidism, and reverse Takotsubo syndrome. While a synergistic interaction is biologically plausible, causality cannot be established from a single observation. Clinically, thyroid function assessment may be considered in COVID-19 patients with cardiovascular symptoms, even with normal myocardial injury markers. COVID-19 may involve multiple systems, including the cardiovascular and neuroendocrine axes, and further research is needed to clarify the potential interactions among viral infection, endocrine disorders, and stress-induced cardiomyopathy.

## Data Availability

The data supporting this case report are included in the article. The raw clinical data of the patient involved are not publicly available due to patient privacy and ethical restrictions but can be shared by the corresponding author upon reasonable request and ethical approval.

## Supplementary material

Supplementary Video S1 Parasternal short-axis (PSAX) views showing left ventricular (LV) regional hypokinesia. LV myocardial thickness is within normal limits. The PSAX view at the mitral valve level shows hypokinesia in the basal inferior wall.

Supplementary Figure S1 Coronary angiography revealed approximately 20% stenosis in the mid-segment of the left anterior descending artery with a myocardial bridge.

Supplementary Figure S2 Resting myocardial perfusion and glucose metabolism imaging showed no significant abnormalities.

Supplementary Figure S3 Echocardiography after 3 years showed normal.
