# Supplementary figures and images for: Takotsubo cardiomyopathy concurrent with hyperthyroidism following COVID-19—a case report and literature review
Source: Front Cardiovasc Med. 2026 Jul 8;13:1862540. doi: 10.3389/fcvm.2026.1862540 (PMC13388277; doi:10.3389/fcvm.2026.1862540)

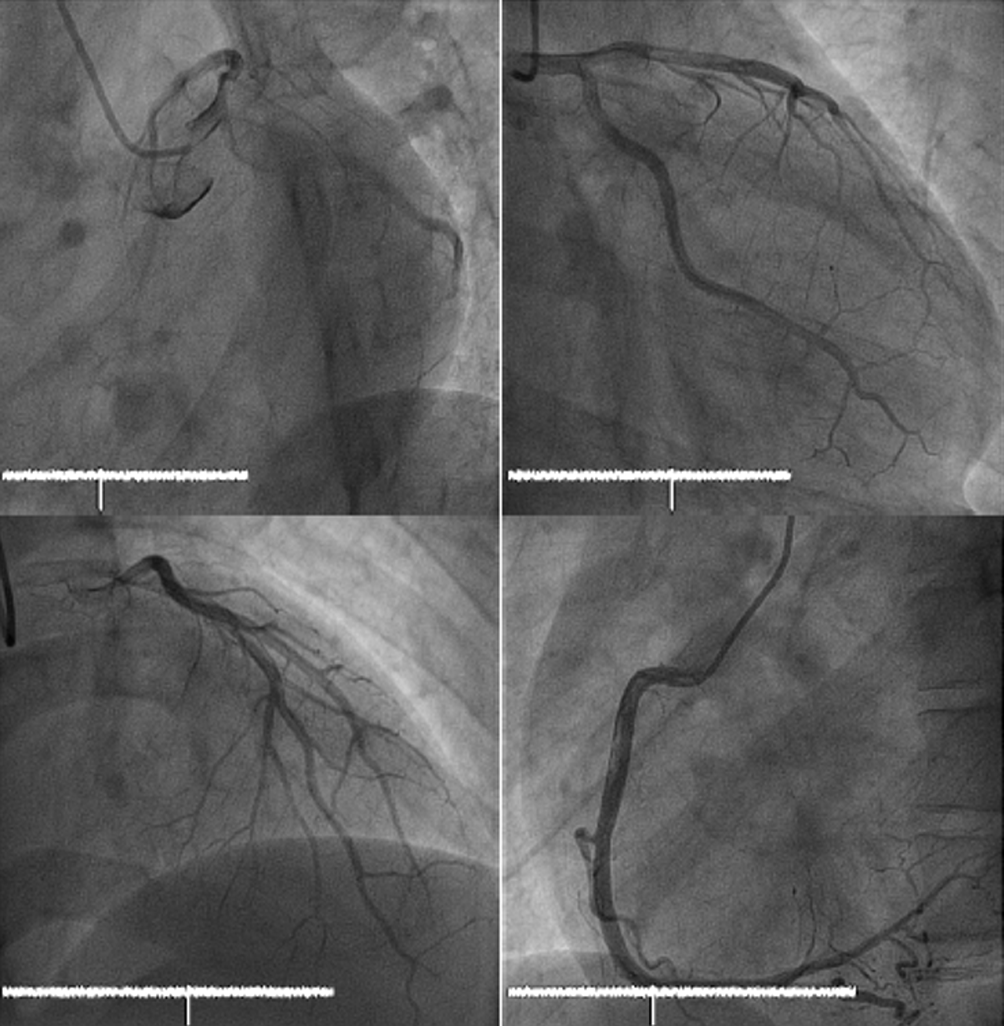

Supplement: Supplementary Figure S1 — Coronary angiography revealed approximately 20% stenosis in the mid-segment of the left anterior descending artery with a myocardial bridge. [file Image1.tif]

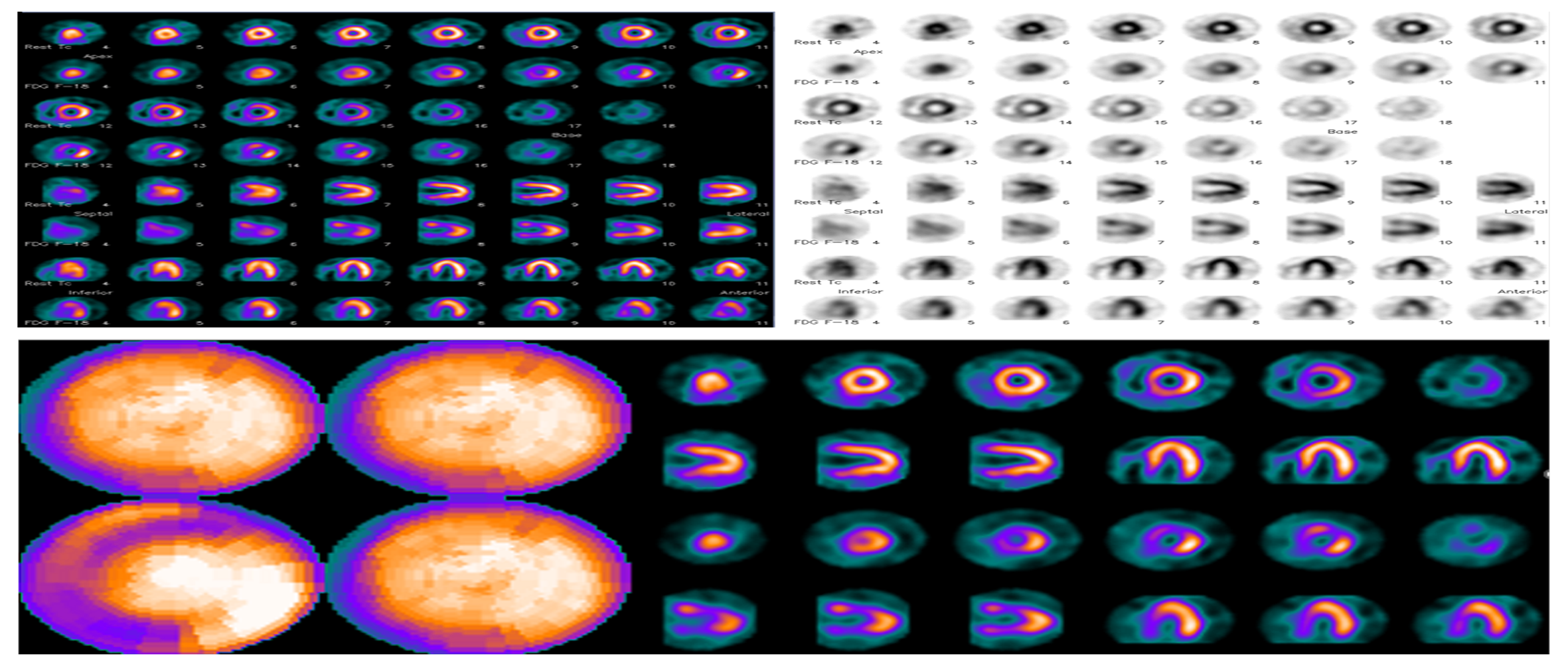

Supplement: Supplementary Figure S2 — Resting myocardial perfusion and glucose metabolism imaging showed no significant abnormalities. [file Image2.tif]

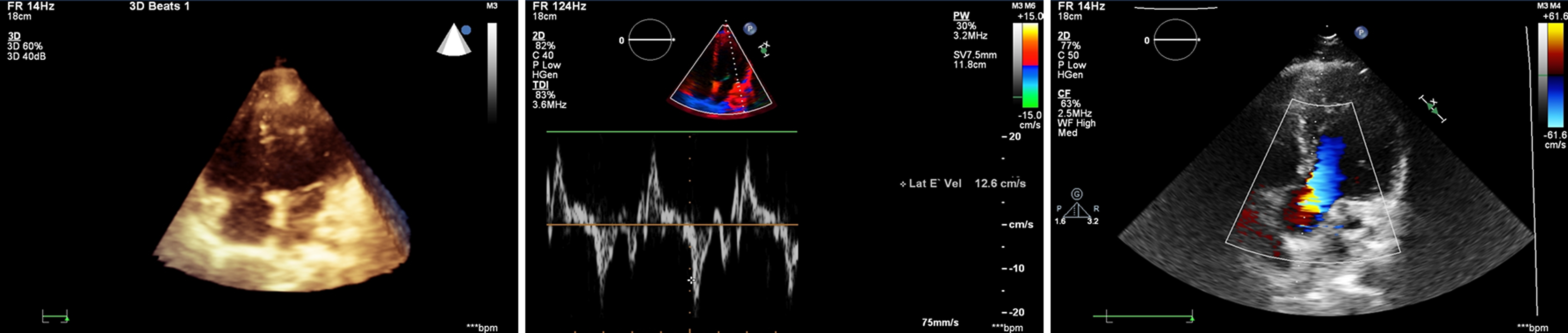

Supplement: Supplementary Figure S3 — Echocardiography after 3 years showed normal. [file Image3.tif]
